# Characterizing the Effects of Focused Ultrasound Therapy in Healthy and Alzheimer’s Disease Neurons

**DOI:** 10.1002/alz.090027

**Published:** 2025-01-09

**Authors:** Amy Shteyman, Rebecca L Noel, Elisa Konofagou

**Affiliations:** ^1^ Columbia University, New York, NY USA

## Abstract

**Background:**

Focused ultrasound (FUS)‐induced blood‐brain barrier opening (BBBO) is a technique for safely, non‐invasively, and transiently opening the blood brain barrier in a targeted area of the brain. Pre‐clinical and clinical studies have shown that FUS is capable of decreasing amyloid plaque load and stimulating neurogenesis in Alzheimer’s Disease (AD) models, in addition to being safe for use in human patients. However, the effect of FUS‐BBBO on neurons has not yet been characterized, despite its crucial role in cognition and regulating brain function. The purpose of the study was to characterize the effects of FUS‐BBBO on neurons in an AD model in order to elucidate the effects of this emerging therapeutic on patients with AD.

**Method:**

In this study, a triple transgenic (3xTg) mouse model of AD was used to investigate the effects of FUS‐BBBO on neurons in progressed AD. The relative expression of 80 key AD genes was compared between 4 different groups: naive wild‐type mice, naive 3xTg mice, FUS‐BBBO treated wild‐type mice, and FUS‐BBBO treated 3xTg mice, with two mice per condition. 2 days post‐FUS‐BBBO, hippocampal neuronal nuclei were isolated for qPCR to quantify relative changes in gene expression between the experimental groups.

**Result:**

In wild‐type mice treated with FUS, synaptic plasticity and neurogenesis genes were over‐expressed. Key AD genes related to amyloid‐beta clearance, neurogenesis, and acetylcholine metabolism began to return to baseline wild‐type values in AD mice treated with FUS in comparison to untreated AD mice.

**Conclusion:**

This study offers preliminary characterization of the effects of FUS in neurons in both WT and AD mice. Future work will help understand the therapeutic effects of FUS BBBO treatment for patients with AD.

